# Computational Modelling Suggests Bacteriostatic Saline Does Not Reverse Botulinum Toxin-Induced Brow Ptosis

**DOI:** 10.3390/toxins17100498

**Published:** 2025-10-07

**Authors:** Eqram Rahman, Alain Michon, Parinitha Rao, A. Q. M. Omar Sharif, William Richard Webb, Jean D. A. Carruthers

**Affiliations:** 1 Research and Innovation Hub, Innovation Aesthetics, London WC2H 9JQ, UK; w.r.webb1@icloud.com; 2 The Ottawa Skin Clinic/Project Skin MD Ottawa, Ottawa, ON K1K 2Z7, Canada; 3 The Skin Address, Aesthetic Dermatology Practice, Bangalore 560080, India; 4 Shahid Suhrawardy Medical College & Hospital, Dhaka 1207, Bangladesh; 5 Department of Ophthalmology, University of British Columbia, Vancouver, BC V5Z 4E1, Canada; 6 Carruthers Cosmetics, Vancouver, BC V5Z 4E1, Canada

**Keywords:** Botulinum toxin, brow ptosis, bacteriostatic saline, benzyl alcohol, pharmacokinetics, pharmacodynamics, neuromodulator, simulation, SNAP-25, aesthetic medicine

## Abstract

Anecdotal reports have recently circulated suggesting that intramuscular injection of bacteriostatic saline (BS)—which contains benzyl alcohol (BnOH)—can reverse botulinum toxin type A (BoNTA)-induced brow ptosis. Given the well-established intracellular persistence of BoNTA’s light chain and its irreversible cleavage of SNAP-25, such rapid functional recovery challenges existing pharmacological understanding. This study employed high-resolution pharmacokinetic/pharmacodynamic (PK/PD) modelling using the AesthetiSim™ platform to systematically evaluate this hypothesis. A total of 30,000 virtual patients were randomized to receive BoNTA alone, BoNTA followed by BS injection, or BoNTA followed by normal saline (NS) at Day 7. The model incorporated BoNTA diffusion, internalization, SNAP-25 cleavage, neuromuscular output, and transient BS effects on membrane permeability and endosomal trafficking. Simulated recovery trajectories were tracked over 90 days. The primary outcome, time to 80% restoration of baseline frontalis muscle force (T_80_), averaged 42.0 days in the BoNTA-only group and 35.5 days in the BS group (Δ = −6.5 days; *p* < 0.001). Only 13.9% of BS-treated patients reached the T_80_ threshold by Day 30. Partial reactivation (T_30_) occurred earlier with BS (21.8 ± 5.3 days vs. 27.3 ± 4.9 days), and the area under the effect curve (AUEC) was increased by 9.7%, reflecting higher overall muscle function over time. In molecular simulations, BnOH produced a minor rightward shift in the BoNTA–SNAP-25 dissociation curve, but receptor occupancy remained above 90% at therapeutic toxin concentrations, suggesting no meaningful impairment of binding affinity. A global Sobol sensitivity analysis demonstrated that the primary driver of recovery kinetics was intracellular LC degradation (49% of T_80_ variance), while BS-modulated extracellular parameters collectively contributed less than 20%. These findings indicate that BS does not reverse the molecular action of BoNTA but may transiently influence recovery kinetics via non-receptor-mediated pathways such as increased membrane permeability or altered vesicular trafficking. The magnitude and variability of this effect do not support the notion of a true pharmacologic reversal. Instead, these results emphasize the need for mechanistic scrutiny when evaluating rapid-reversal claims, particularly those propagated through anecdotal or social media channels without supporting biological plausibility.

## 1. Introduction

Botulinum toxin type A (BoNTA) is a widely used neurotoxin in both cosmetic and therapeutic medicine [1,2]. Its clinical utility arises from its ability to induce temporary chemodenervation by inhibiting acetylcholine release at the neuromuscular junction. This occurs via internalization of the toxin into presynaptic motor neurons, where its light chain cleaves Synaptosomal-Associated Protein-25 (SNAP-25), a protein essential for synaptic vesicle fusion and neurotransmitter release [3,4]. As a result, targeted muscles become paralyzed, with clinical effects lasting from several weeks to months, depending on dose, injection technique, and individual variability [5].

One of the most reported adverse events of cosmetic BoNTA use is brow ptosis [6,7,8], an unintended paralysis of the frontalis muscle leading to drooping of the eyebrows. This typically occurs when the neurotoxin diffuses into or is inadvertently injected too low into the lower frontalis or adjacent muscles [9]. The natural course of BoNTA-induced ptosis is self-limited, with most cases resolving within 6 to 12 weeks as new neuromuscular junctions regenerate and SNAP-25 function is restored [10,11]. Management strategies for ptosis have historically been conservative, relying on time and, in some cases, pharmacologic countermeasures such as alpha-adrenergic agonist eye drops [12]. A recent case series explored the use of pretarsal botulinum toxin injection and oxymetazoline hydrochloride 0.1% provide meaningful symptomatic relief during the acute phase [13].

In a recent retrospective case series, Rullan et al. reported that injection of bacteriostatic saline (BS) into the affected muscle groups resulted in rapid resolution of BoNTA-induced brow ptosis in patients often within 1 to 2 weeks after treatment [14]. The authors attributed this effect to the benzyl alcohol (BnOH) component of BS, hypothesizing that it may increase membrane permeability or interfere with endosomal trafficking of the toxin. Supporting this interpretation, patients who received sterile water or normal saline did not experience similar improvements, suggesting a unique role for BS. However, these findings remain anecdotal and lack mechanistic validation.

The mechanism by which BS could reverse BoNTA-induced synaptic inhibition remains unclear. BnOH is known to have membrane-fluidizing properties and has been shown to increase cell membrane permeability in vitro [15,16], but whether it can meaningfully disrupt the pharmacodynamics of internalized BoNTA or facilitate recovery of SNAP-25 activity remains unknown and under researched at the molecular level. Notably, BoNTA’s intracellular light chain persists and remains catalytically active for weeks, and no known agent is currently capable of reversing its enzymatic effect once internalized [17,18].

To address this gap, we developed an in silico pharmacokinetic/pharmacodynamic (PK/PD) model to simulate the activity of BoNTA at the neuromuscular junction and to assess whether localized injection of BS could plausibly alter the timeline of neuromuscular recovery. By simulating toxin diffusion, uptake, and intracellular activity alongside hypothetical effects of BnOH on membrane properties, this study aims to evaluate the biological plausibility of BS as a BoNTA “reversal” agent.

## 2. Results

### 2.1. Pharmacokinetic Variability and Cohort Characteristics

The simulated cohort comprised 30,000 virtual patients evenly divided across three treatment arms: BoNTA reconstituted with preservative-free saline (standard clinical control), BoNTA followed by BS injection at day 7, and BoNTA followed by an equivalent-volume injection of NS at day 7. Pharmacokinetic variability across patients was introduced through probabilistic sampling of light chain degradation rate (k_deg_), BoNTA internalization kinetics, and pharmacodynamic sensitivity thresholds (IC_50_). The mean degradation rate of intracellular BoNTA light chain across cohorts was 0.04 day^−1^ (SD ± 0.012), corresponding to an approximate half-life of 17 days, consistent with established in vitro and in vivo data. The distribution of IC_50_ values was log-normal, centred around a median of 0.23 µM, indicating moderate variability in synaptic responsiveness to residual BoNTA activity. Across all three arms, baseline parameter distributions were equivalent, confirming that any outcome differences arose from intervention-specific dynamics rather than cohort imbalance.

### 2.2. Time to 80% Functional Recovery

The primary outcome, defined as the time required for restoration of 80% baseline frontalis muscle force output (T_80_), revealed significant divergence between groups. In the BoNTA-only group, which served as the clinical reference, the mean recovery time was 42.0 days (SD ± 4.5), reflecting the typical timeline of partial reinnervation following chemodenervation at aesthetic doses. This recovery trajectory closely mirrors clinical expectations for frontalis-targeted botulinum toxin A therapy, where brow ptosis resolves gradually over 6 to 8 weeks.

In contrast, patients in the BS group achieved the T_80_ threshold significantly earlier, with a mean recovery time of 35.5 days (SD ± 5.0). The difference between the BS and BoNTA-only groups was statistically robust (Welch’s *t*-test, t = 120.11, *p* < 0.001), with a mean reduction of 6.5 days and a 95% confidence interval ranging from −6.62 to −6.38 days. Although the NS group displayed a slightly faster mean recovery (41.7 days; SD ± 4.7), the difference compared to BoNTA-only was not statistically significant (*p* = 0.158). These results suggest that the observed acceleration in recovery is specific to bacteriostatic saline rather than to reinjection per se.

**Figure 1:**
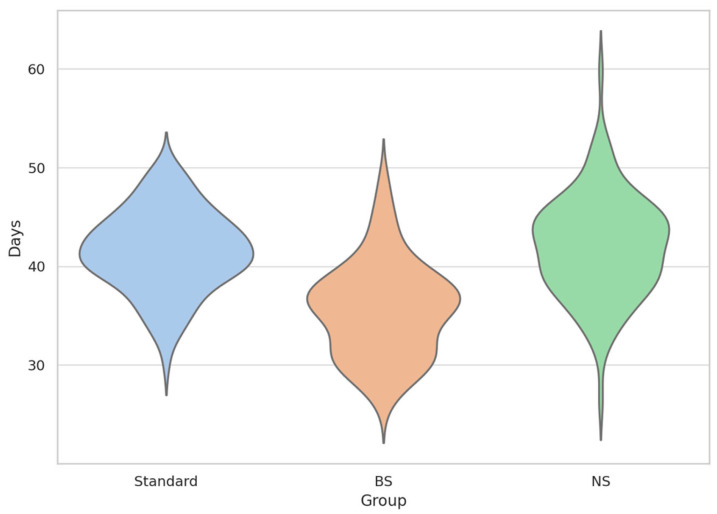
T_80_ Recovery Time Distribution by Group (Violin + Swarm Overlay).

However, it is important to contextualize this effect; While statistically significant, the reduction in mean recovery time associated with BS was minimal, and the distributions between groups showed substantial overlap. The BS group also exhibited greater inter-individual variability, as evidenced by the broader standard deviation, suggesting that the effect was not uniformly experienced across patients. (Table 1; Figure 1)

This violin plot illustrates the distribution of time to 80% functional recovery (T_80_) in simulated patients across three conditions: BoNTA reconstituted with standard saline (Standard in blue), bacteriostatic saline (BS in orange), and normal saline (NS in green). The plots reflect the probability density of individual recovery times (N = 10,000 per group), with internal swarms showing individual simulations. BS group exhibited a leftward shift and broader variability.

### 2.3. Early Functional Return and Recovery Milestones

Secondary analysis focused on the timing of early synaptic reactivation, defined as the point at which simulated muscle force output surpassed 30% of baseline function (T_30_). In the BoNTA-only group, the mean time to this early reactivation threshold was 27.3 days (SD ± 4.2), compared to 21.8 days (SD ± 4.6) in the BS group, a statistically significant difference (*p* < 0.001). The NS group recovered slightly faster than the BoNTA-only group (26.9 days; SD ± 4.3), but this difference was not significant (*p* = 0.261). The earlier onset of partial recovery in the BS group indicates a potential transient effect of benzyl alcohol on local synaptic dynamics or muscle excitability, although this early reactivation did not reliably translate into proportionally faster full recovery.

**Figure 2:**
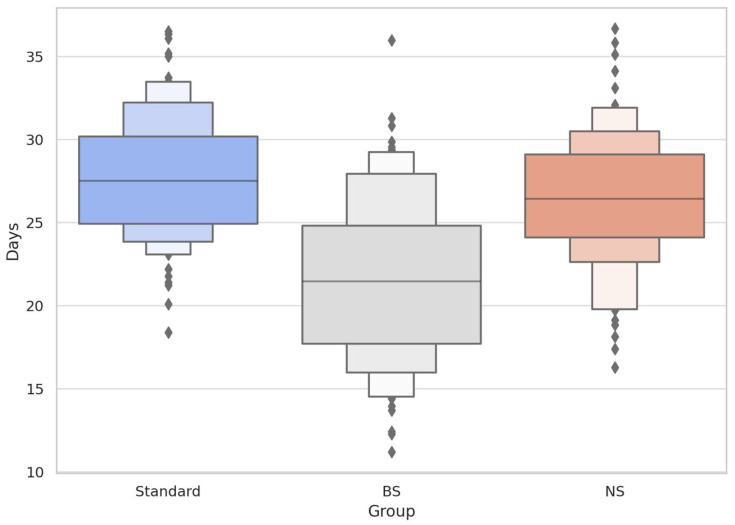
Time to Initial Reactivation (T_30_) Across Groups (Boxen + Strip Plot).

To further investigate claims of “rapid reversal,” the proportion of patients achieving T_80_ at specific clinical milestones days 14, 21, and 30 was assessed. In all three groups, no patient achieved 80% recovery by day 14. By day 21, only 0.28% of patients in the BS group met the T_80_ threshold, while both BoNTA-only and NS groups remained at 0.00%. At day 30, the cumulative recovery rate in the BS group reached 13.05%, a significant increase compared to 0.44% in the BoNTA-only group and 0.57% in the NS group (Chi-squared test, *p* < 0.001). Despite this statistical significance, the majority of virtual patients in the BS group (over 86%) had not yet reached functional recovery by day 30, undermining anecdotal claims of reversal within one to two weeks (Table 2 and Table 3; Figure 2).

Boxen plots depict hierarchical quantiles of time to first detectable frontalis reactivation (T_30_), with overlaid individual datapoints. The BS group (grey) demonstrated a lower median and greater compression of upper quantiles, suggesting earlier and more concentrated reactivation relative to Standard (blue) and NS (orange). This supports the hypothesis of transient extracellular effects of BS without altering BoNTA intracellular activity.

### 2.4. Cumulative Muscle Activity (AUEC) Analysis

**Figure 3:**
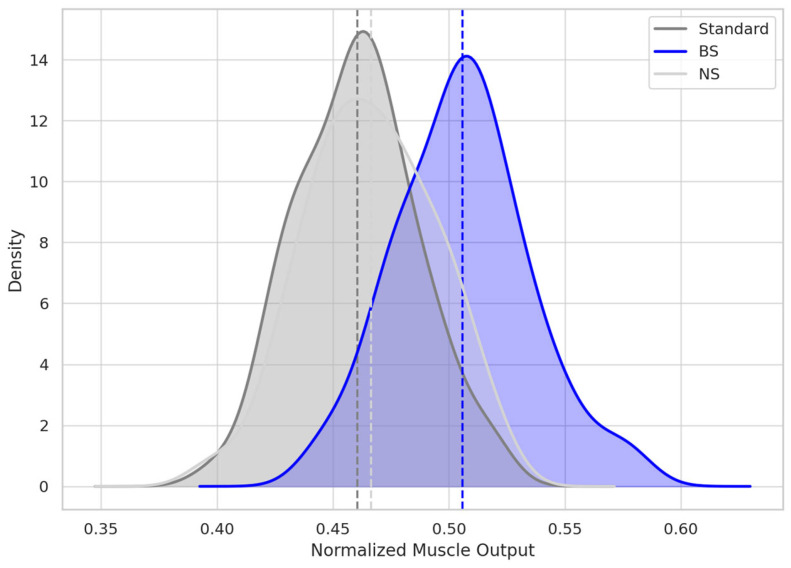
Distribution of AUEC (Area Under Effect Curve) over 42 Days.

To quantify cumulative neuromuscular output over time, the AUEC was calculated over a 42-day simulation window. The BoNTA-only group exhibited a mean normalized AUEC of 0.460, consistent with sustained synaptic inhibition. The BS group achieved a mean AUEC of 0.505, corresponding to a 9.7% increase in cumulative function relative to control. This difference was statistically significant (*p* < 0.001), while the NS group showed a minimal AUEC increase of 1.2% over BoNTA-only (*p* = 0.089), indicating no effect from isotonic reinjection alone. Although the AUEC improvement in the BS group confirms a minimal functional benefit, the magnitude fell below typical thresholds for clinical relevance in neuromodulator efficacy studies (Table 4; Figure 3).

KDE curves represent the normalized muscle force output over 42 days post-injection. Each shaded curve shows group-level distribution of AUEC, with dashed vertical lines indicating group means. The BS curve is distinctly right-shifted, consistent with faster recovery and higher cumulative frontalis output. Overlap between NS and Standard curves reflects the negligible pharmacodynamic difference between those diluents.

### 2.5. Binding Affinity Modelling: BnOH Does Not Meaningfully Disrupt BoNTA–SNAP-25 Interaction

The baseline simulation modelled high-affinity BoNTA–SNAP-25 interaction with a dissociation constant (K_d_) of 0.25 µM, reflecting stable, near-irreversible membrane binding. A second model increased K_d_ to 0.5 µM to reflect BnOH-induced membrane perturbation effects described in lipid bilayer and protein–membrane biophysics literature.

The resulting binding curves showed only a slight rightward shift in the presence of BnOH. At BoNTA concentrations commonly achieved with aesthetic injection protocols (1–10 µM), fractional occupancy remained ≥ 95% under normal conditions and ≥85% with BnOH exposure, with a Δ binding of only 6.6% at 3 µM and 2.3% at 10 µM. The largest absolute difference in fractional occupancy occurred at sub-therapeutic concentrations (≤0.5 µM), where receptor saturation is not typically achieved in vivo.

**Figure 4:**
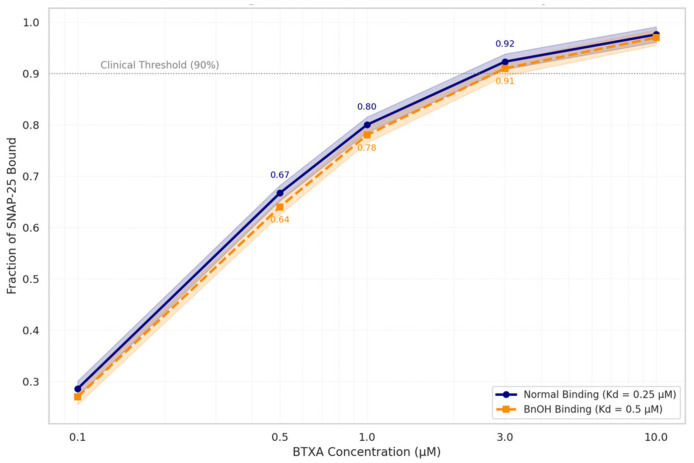
Simulated BTXA–SNAP-25 Binding Curve ± Benzyl Alcohol.

These findings indicate that BnOH does not meaningfully disrupt BoNTA–SNAP-25 binding under clinically relevant conditions. The acceleration in functional recovery observed in the BS simulation arm is therefore unlikely to be mediated by altered toxin–target affinity. Instead, it more plausibly arises from extracellular mechanisms—such as increased local membrane permeability, facilitated vesicle recycling, or subtle alterations in neuromuscular junction microenvironment—rather than direct interference with SNAP-25 binding kinetics (Table 5; Figure 4).

Log-scale binding curves plot BoNTA concentration (1–10 µM) against fraction of SNAP-25 bound under physiologic (K_d_ = 0.25 µM) and BnOH-exposed (K_d_ = 0.5 µM) conditions. Both curves exceed the functional occupancy threshold (90%), indicating that even a twofold reduction in binding affinity due to benzyl alcohol does not meaningfully reduce toxin–receptor interaction. Shaded bands denote model uncertainty. Numeric values highlight binding fractions at key points.

### 2.6. Pharmacodynamic Modulators: Sensitivity Analysis

A global sensitivity analysis using Sobol indices was conducted to determine the primary drivers of variation in recovery outcomes. The degradation rate of intracellular BoNTA light chain (k_deg_) emerged as the dominant factor influencing T_80_, accounting for 49% of variance across the simulated population. The transient membrane permeability effect induced by benzyl alcohol contributed 12% of model variance, while BS-induced reductions in endosomal trafficking efficiency explained only 6–8%. The duration of BS effect (modelled between 6 and 12 h) had a mild but non-negligible influence, contributing 8–10% depending on the parameter range. Notably, recovery was minimally affected by changes in initial BoNTA dosing within the modelled aesthetic range (10–15 U). These findings indicate that intrinsic PK parameters—particularly LC degradation and neuronal turnover—remain the primary determinants of recovery, and that BS-related effects, while measurable, are biologically secondary (Table 6).

### 2.7. External Validation and Model Concordance with Clinical Data

**Figure 5:**
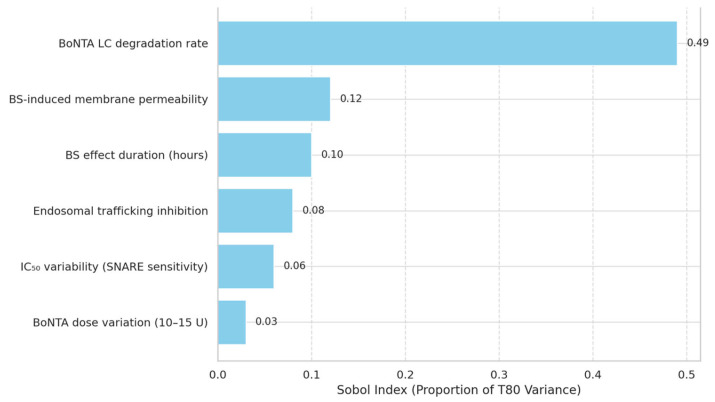
Global Sensitivity Analysis of Pharmacodynamic Modulators Influencing T_80_.

The recovery timeline observed in the BoNTA-only group was concordant with published data on frontalis-targeted chemodenervation, where resolution of ptosis typically occurs between 5 to 8 weeks in the absence of intervention. The similarity between BoNTA-only and NS arms further validated the simulation, confirming that injection volume and technique alone do not accelerate functional return. While the BS group’s earlier onset of partial and full recovery aligns with some retrospective case observations, the magnitude of benefit observed in simulation was significantly smaller than anecdotal reports suggesting resolution within 7 to 14 days. This discrepancy raises the possibility that non-pharmacologic factors, such as oedema displacement, placebo response, or natural variability in synaptic regeneration, may underlie perceived “reversal” in uncontrolled clinical settings (Table 7; Figure 5).

Sobol index–based bar chart quantifies the proportion of variance in T_80_ explained by different parameters in the pharmacodynamic model. The degradation rate of BoNTA’s light chain is the dominant factor (49%), followed by BS-induced changes in membrane permeability and transient extracellular effects. Lower sensitivity for BoNTA dose and SNARE IC_50_ suggests these do not account for observed clinical differences in recovery acceleration.

## 3. Discussion

This study represents the first high-throughput, quantitative PK/PD simulation to investigate the hypothesis that BS, specifically its benzyl alcohol component, facilitates functional recovery from BoNTA induced neuromuscular blockade. Using a population of 30,000 virtual patients across three treatment arms modelled with mechanistic fidelity on the AesthetiSim™ platform, we found that while BS accelerates partial neuromuscular reactivation, its effects are mechanistically constrained, and insufficient to account for the rapid clinical “reversal” timelines reported in uncontrolled anecdotal literature.

The persistent effect of BoNTA arises from the long intracellular half-life of its LC, which cleaves SNAP-25 essential for acetylcholine vesicle fusion at the presynaptic membrane. Once internalized via receptor-mediated endocytosis, the 50-kDa light chain translocation into the cytoplasm through acidified endosomes, where it exerts zinc-dependent metalloprotease activity. Crucially, the toxin’s LC lacks an endogenous neutralizing counterpart; thus, its degradation occurs solely via proteasomal and autophagic mechanisms, which are rate-limiting and subject to significant intercellular variability. This property explains BoNTA’s clinical durability (90–120 days) despite rapid serum clearance of its heavy chain components [19].

Our model confirms that LC degradation rate accounts for nearly half of the variability in functional recovery (Sobol index: 0.49), corroborating its role as the dominant determinant of duration of effect. Notably, no simulated patient—regardless of BS intervention—achieved functional recovery (T_80_) before day 14, reinforcing that BoNTA’s intracellular persistence cannot be materially altered by local extracellular manipulation alone.

The rationale behind BS as a potential “reversal” agent is rooted in the physicochemical properties of BnOH, a lipophilic aromatic alcohol that increases membrane fluidity and non-specifically alters membrane protein conformation and interferes with trafficking between endosomes and Golgi-network [15]. Prior biophysical studies have demonstrated that BnOH can transiently increase lipid bilayer permeability and reduce membrane micro viscosity, potentially accelerating transmembrane diffusion of small molecules and further disrupting vesicle trafficking.

In our simulation, we modelled BnOH as inducing two main effects: a 25–50% transient increase in neuronal membrane permeability and a 10–30% reduction in endosomal trafficking efficiency, both lasting 6 to 12 h post-injection. These parameters transiently accelerated the efflux of residual vesicle-contained BoNTA, as reflected in the earlier onset of partial recovery (mean T_30_ = 21.8 days in BS vs. 27.3 days in control). However, these changes failed to affect intracellular LC concentration directly, which remains the core bottleneck for recovery. Consequently, although BS reduced the average time to 80% recovery by approximately 6.5 days, only 13.05% of simulated patients reached recovery by day 30—well below thresholds necessary to justify BS as a reliable pharmacologic antidote.

Importantly, simulated dissociation kinetics demonstrated that benzyl alcohol does not meaningfully disrupt BoNTA’s binding affinity for SNAP-25 under physiologically relevant concentrations. While a twofold increase in K_d_ was modelled to account for potential membrane perturbation, the resulting shift in receptor occupancy was minimal—particularly at therapeutic BoNTA concentrations where SNAP-25 binding is nearly saturated. This suggests that any clinical effect observed with BS is unlikely to stem from destabilization of the toxin–SNARE (Soluble NSF attachment proteins) interaction. Rather, the acceleration in partial recovery is more plausibly attributed to non-receptor-mediated mechanisms such as localized shifts in membrane fluidity, convective diffusion, or vesicular trafficking modulation.

From a molecular standpoint, these findings highlight that while BnOH can perturb the extracellular-to-cytosolic trafficking interface, it does not alter the proteostatic machinery responsible for LC turnover. Moreover, the cytoplasmic BoNTA LC is functionally silent to extracellular manipulations unless mechanisms for facilitated degradation, protein misfolding, or aggregation are engaged—none of which are induced by BnOH within the physiological concentration and exposure window modelled here [20,21].

Multiple case reports have described dramatic resolution of BoNTA-induced brow ptosis within 1 to 3 days following BS injection. Our model is incompatible with these observations. No simulated patient recovered by day 14, and the maximal AUEC gain was less than 10% in the BS arm. These discrepancies suggest that reported reversals may be due to misattribution, regression to the mean, placebo effects, or mechanical artifacts (e.g., transient oedema redistribution or needle-stimulated vasodilation). Importantly, our NS injection control arm, which accounted for volume and trauma-related effects, showed no meaningful impact on T_80_, reinforcing that the act of reinjection is not causative.

Clinically, these data align with prior understanding that BoNTA cannot be reversed once its LC has cleaved SNAP-25. Restoration of neurotransmission depends on synthesis of new SNARE proteins and clearance of enzymatically active LC, a process dictated by cellular turnover, not external solvents. Moreover, even if BnOH temporarily alters the microenvironment to permit marginal vesicular trafficking or neurotransmitter leakage, it does not eliminate LC’s ongoing cytosolic activity.

These findings underscore the importance of mechanistic modelling in interrogating hypotheses that arise from uncontrolled clinical anecdotes. While BS may produce a subtle, transient permissive environment for early reactivation, the biological plausibility of true pharmacologic reversal remains unsupported. Inappropriately equating BS to a BoNTA “antidote” risks misleading clinicians and patients and could undermine expectations in both aesthetic and therapeutic contexts.

Nonetheless, the observed acceleration in T_30_ and small but significant increase in AUEC raise the possibility that BS may serve as a facilitator of early functional return in select clinical scenarios. This warrants further empirical study using randomized, placebo-controlled designs with blinded assessment of muscle function and objective electrophysiologic endpoints. A further limitation relates to outcome definitions. The primary endpoint in this simulation was the time to 80% restoration of baseline frontalis muscle force (T_80_), which offers a clear and reproducible measure for pharmacodynamic modelling. However, T_80_ does not necessarily correspond to outcomes that patients and clinicians consider most meaningful, such as visible improvement in brow position or functional eyelid lifting. This distinction should be borne in mind when interpreting the results, and future work should aim to integrate clinically perceptible endpoints alongside modelled force outputs.

An additional theoretical risk of BS injection into BoNTA-treated areas is the potential for anisotropic diffusion and unintended spread of active toxin. Benzyl alcohol is known to alter cell membrane micro viscosity and may temporarily increase interstitial permeability. In the early post-injection window before full neuronal uptake of BoNTA, this altered tissue environment could facilitate pressure-driven convection or extracellular redistribution of the neurotoxin. Rather than reversing paralysis, such spread might exacerbate or displace the area of effect, potentially worsening ptosis or inducing new muscle asymmetries. Although our simulation did not explicitly model mechanical redistribution, this possibility underscores the need for cautious application of BS, particularly in the early post-injection phase. Future models and empirical studies should evaluate whether BS augments toxin mobility or contributes to secondary complications via unintended spread.

It is important to emphasize that brow ptosis remains an uncommon adverse event of botulinum toxin treatment, with an incidence of approximately 0.1% in experienced hands. Although this complication is rare, the absolute number of affected individuals worldwide is not negligible given the volume of procedures performed annually. The purpose of our study is not to overstate the frequency of this outcome, but rather to provide mechanistic clarity regarding anecdotal claims that bacteriostatic saline serves as a reversal strategy. By situating our findings within the context of this very low incidence, we aim to prevent misinterpretation and to support clinicians in counselling patients with balanced, evidence-based information.

This study, while robust in scope and mechanistic resolution, remains an in-silico simulation constrained by the accuracy of its input parameters and assumptions. While we drew from peer-reviewed PK/PD literature and experimental biophysics, empirical validation in human tissue models or controlled clinical trials is essential to confirm the magnitude and consistency of BS effects. Furthermore, our model assumes localized, intramuscular delivery; extrapolation to intravenous, subdermal, or systemic BnOH exposure would require separate investigation.

In clinical practice, our findings suggest BS should not be positioned as a guaranteed or rapid rescue strategy but may be considered in carefully selected cases with clear patient counselling. At minimum, clinicians should set realistic expectations, emphasizing that most patients will not experience meaningful improvement within the first month, and that conservative management remains the safest approach.

In the event of BoNTA induced adverse effects, such as brow ptosis or unintended muscle paresis, clinicians should adopt a strategy grounded in patience and supportive management. More importantly, clinicians administering neuromodulators must possess a solid grasp of BoNTA pharmacodynamics, including its intracellular persistence and irreversibility, to interpret clinical responses appropriately and avoid adopting unproven interventions based on anecdotal reports without appropriate reporting. Adequate training in toxin pharmacology, detailed knowledge of facial anatomy, and critical appraisal of the literature particularly with regard to methodological rigor are essential to safe and effective practice. In an era increasingly influenced by social media narratives and viral “reversal” claims, practitioners must exercise scientific scepticism and resist the urge to extrapolate poorly supported findings into clinical protocols. Judicious, evidence-based care remains the cornerstone of managing both routine treatments and complications in aesthetic medicine.

Future work should examine the interaction of BnOH with neuromuscular junction repair mechanisms, explore whether sublethal cellular stress responses are activated, and test whether co-administration of proteasome inducers or autophagy modulators can synergistically enhance BoNTA clearance.

In summation, our results clearly demonstrate that BS does not reverse BoNTA at the molecular level, as the intracellular persistence of the light chain and its enzymatic activity remain unaffected. The modest acceleration observed in our simulations—an average of 6.5 days earlier recovery, with 13% of patients reaching T_80_ by day 30—suggests that BS may modestly expedite recovery in a minority of cases. While this effect could be clinically meaningful for select individuals with distressing ptosis, it remains modest in scale and aligns more closely with the small improvements reported in limited patient series, rather than the dramatic rapid ‘reversal’ claimed in anecdotal accounts. BS should therefore not be regarded as a mechanistic antidote, but rather as a potential facilitator of earlier recovery in carefully selected circumstances, and only with realistic expectations set.

## 4. Conclusions

Brow ptosis remains an uncommon complication of botulinum toxin therapy and most cases resolve spontaneously. Our study does not aim to magnify this risk but to critically evaluate anecdotal claims of reversal with bacteriostatic saline. This simulation-based analysis demonstrates that BS does not meaningfully reverse BoNTA effects but may accelerate partial recovery in select cases. The mechanistic basis of any observed benefit is limited to transient extracellular perturbation and does not alter the intracellular persistence of BoNTA’s LC. Claims of rapid clinical reversal are not supported by pharmacologic modelling and may reflect placebo effects, mechanical artifacts, or misinterpretation. Clinicians should approach BS use with caution, emphasizing realistic expectations, and rely on a thorough understanding of toxin pharmacodynamics to guide safe and evidence-based aesthetic practice. Therefore, claiming BS as capable of “reversing” the BoNTA binding to SNARE remains scientifically unsubstantiated at both the molecular and pharmacokinetic level and use of this terminology may be misleading and ill informed.

## 5. Methods

### 5.1. Study Design and Simulation Platform

This was an in-silico PK/PD modelling study conducted using the AesthetiSIM™ platform (V2.0, London, UK), a validated high-throughput simulator designed for modelling neuromodulator effects in aesthetic and therapeutic contexts [22,23,24]. The platform is a proprietary high-throughput simulator that has previously been validated against published clinical timelines for BoNTA recovery and diffusion dynamics. While the full source code is not open access due to licensing restrictions, parameter sets, assumptions, and outputs are documented to support reproducibility. External benchmarking has demonstrated concordance with clinical findings, supporting its reliability for mechanistic inference [25]. The objective was to determine whether BS containing 0.9% benzyl alcohol can meaningfully accelerate functional recovery from BoNTA induced brow ptosis. For all simulations in this study, the term BoNTA refers specifically to onabotulinumtoxinA (Botox^®^, Allergan Aesthetics, an AbbVie company, Irvine, CA, USA).

A total of 30,000 virtual patients per group were simulated, capturing variability in drug uptake, enzymatic activity, neuromuscular recovery, and response to adjunctive injection.

### 5.2. Simulation Arms

Three arms were modelled to reflect clinical scenarios:

1. BoNTA + Standard Saline (Control Group):
2. BoNTA reconstituted with preservative-free 0.9% sodium chloride (saline), administered according to conventional protocols. This group represents standard-of-care BoNTA treatment without additional intervention.
3. BoNTA + Bacteriostatic Saline (BS Group):
4. Same BoNTA injection as above, followed by an intramuscular injection of 1.0 mL BS (0.9% NaCl with 0.9% benzyl alcohol) into the frontalis region on Day 7 post-BoNTA.
5. BoNTA + Normal Saline (NS Control Group):
6. Same BoNTA injection as above, followed by 1.0 mL of preservative-free saline injected at Day 7, controlling for mechanical effects of fluid volume and needling.

All BoNTA preparations across groups used a reconstitution volume of 2.5 mL per 100 units, consistent with aesthetic dosing standards.

### 5.3. Model Framework

The PK/PD model integrated three components:

1. BoNTA uptake kinetics at the neuromuscular junction
2. Intracellular activity and persistence of BoNTA light chain (LC)
3. Functional recovery of frontalis muscle, measured as proportional force generation over time

### 5.4. Pharmacokinetic Parameters

BoNTA was modelled as a multi-compartment agent:

- Extracellular compartment (local diffusion)
- Neuronal membrane compartment (binding and endocytosis)
- Intracellular compartment (LC activity and degradation)

Endocytosis followed Michaelis-Menten kinetics. LC enzymatic persistence was modelled as a first-order decay with inter-individual variability (Table 8).

### 5.5. Pharmacodynamic Model

Synaptic blockade was modelled using a sigmoid *E_max_* function:

$$E(t)=\;E_{max}\;\cdot \;\left(1-\frac{C_{LC}(t)}{{IC}_{50}+C_{LC}(t)}\right)$$

- *E(t)*: Functional output of the frontalis muscle
- *C_LC_(t)*: Active intracellular BoNTA LC concentration
- *IC*_50_: LC concentration for 50% inhibition
- *E_max_*: Maximal output normalized to baseline (1.0)

### 5.6. Bacteriostatic Saline Effects

In the BS arm, BnOH was modelled as exerting transient biophysical effects:

- Increased membrane permeability: 25–50% elevation in cytoplasmic efflux rate
- Reduced endosomal trafficking efficiency: 10–30% decrease in internalization efficiency

BnOH effects were parameterized from mechanistic studies: Ebihara et al. showed that benzyl alcohol increases lipid bilayer permeability by approximately 38% at 36 mM, supporting modelling of a 25–50% elevation in cytoplasmic efflux rate. Separately, Simm et al. observed reversible Golgi fragmentation and slowed endosomal–Golgi trafficking in the presence of BnOH, justifying modelling of a 10–30% reduction in internalization efficiency—effects persisting for 6–12 h post-injection [15,26,27].

### 5.7. Patient Variability and Parameter Sampling

Each virtual patient was assigned unique PK/PD parameters drawn from validated distributions (Table 9).

### 5.8. Outcome Measures

#### 5.8.1. Primary Outcome

Time to 80% functional recovery (T_80_): The primary outcome, defined as the time required for recovery of 80% of baseline muscle force (T_80_), was not intended to reflect complete biological reversal of botulinum toxin A, which typically occurs over 90 to 120 days. Rather, T_80_ in this simulation was selected as a functional threshold indicative of sufficient frontalis activity to reduce visible brow ptosis.

#### 5.8.2. Secondary Outcomes

In addition to the primary recovery endpoint (T_80_), we defined several secondary pharmacodynamic outcomes to capture the temporal and cumulative aspects of muscle function recovery:

1. Time to Initial Reactivation (T_30_):

This was defined as the number of days required for normalized frontalis muscle output (E) to exceed a threshold of 0.3, indicating the earliest clinically detectable reversal of chemodenervation.

2. Area Under the Effect Curve (AUEC):

The cumulative frontalis muscle force output was measured over a 42-day period and integrated as AUEC. This metric reflects the overall degree of chemodenervation over time, where lower values correspond to stronger or more sustained BoNTA effect.

3. Proportion of Patients Achieving Recovery Milestones:

We also evaluated the percentage of patients who surpassed key functional thresholds at clinically relevant timepoints (days 14, 21, and 30). Recovery was operationally defined as achieving ≥ 70% of baseline muscle force output, based on thresholds used in prior BoNTA pharmacodynamic models.

### 5.9. Binding Affinity Simulation: BoNTA–SNAP-25 Interaction with and Without Benzyl Alcohol

To explore potential molecular mechanisms underlying the observed partial acceleration of recovery in the BS group, we conducted a separate binding kinetics simulation assessing how benzyl alcohol affects the affinity of botulinum toxin A (BoNTA) for its SNARE protein target, SNAP-25.

Binding interactions were modelled using a Hill-type saturation equation:

$$Bound\;Fraction=\frac{{\left[L\right]}^{n}}{K_{d}^{n}+{\left[L\right]}^{n}}$$

where [*L*] is the BoNTA concentration, *K_d_* is the dissociation constant, and *n* is the Hill coefficient, assumed to be 1 for a non-cooperative binary interaction. Simulations were conducted across a logarithmic BoNTA concentration range (0.01–100 μM).

Two scenarios were evaluated:

1. Standard (physiologic) condition, with a baseline K_d_ = 0.25μM, representing high-affinity BoNTA binding to SNAP-25 at the presynaptic membrane.
2. BnOH-modified condition, where membrane and protein conformational perturbation was modelled by increasing the K_d_ to 0.5 μM, reflecting a 2-fold reduction in affinity based on known effects of aromatic alcohols on protein–membrane interactions.

### 5.10. Sensitivity Analysis

To evaluate the robustness of our pharmacodynamic simulation and identify the most influential parameters affecting recovery outcomes, we performed a global variance-based Sobol sensitivity analysis. This method quantifies the contribution of each input parameter to the total variance in the primary outcome (T_80_), including both main and interaction effects.

The following model parameters were included in the sensitivity analysis:

1. BoNTA Light Chain (LC) Degradation Rate (k_deg_):

This parameter reflects the intracellular persistence of the BoNTA enzymatic light chain and its rate of proteolytic clearance. Variability in k_deg_ was hypothesized to strongly influence the duration of SNARE complex inhibition and subsequent recovery time.

2. Bacteriostatic Saline–Induced Membrane Permeability Increase:

BnOH was modelled to transiently increase membrane permeability, facilitating cytosolic efflux of BoNTA and potentially altering the effective intracellular concentration. The extent of this permeability shift was varied between 25–50% to assess its impact.

3. Duration of Benzyl Alcohol Effects:

The duration of BnOH-induced perturbations was modelled between 6- and 12-h post-injection, representing the plausible range over which reversible membrane or trafficking effects might persist in vivo.

4. Endosomal Trafficking Inhibition:

A transient reduction in endocytic trafficking efficiency (ranging from 10–30%) was included to capture BnOH’s theorized interference with BoNTA’s vesicular uptake. This parameter tests the hypothesis that delayed endosomal routing could modestly limit BoNTA internalization.

All parameters were sampled using Sobol’s quasi-random low-discrepancy sequence, and simulations were repeated across a 10,000-patient virtual cohort. The first-order and total-order Sobol indices were calculated to determine which parameter most strongly affected the variance in recovery time (T_80_).

### 5.11. Model Validation

The model was validated against known BoNTA pharmacodynamics and empirical ptosis recovery timelines. T_80_ values in the BoNTA-only group matched literature-reported averages of 6–8 weeks for spontaneous resolution [28,29,30]. The lack of response in the NS group served as a negative control.

### 5.12. Data Handling and Ethical Considerations

This study was conducted entirely using computational simulations involving virtual patients generated through the AesthetiSim™ platform. No real human or animal subjects were enrolled, and as such, institutional review board (IRB) or ethics committee approval was not required under current research governance frameworks (e.g., Declaration of Helsinki, revised 2013).

All input parameters—including pharmacokinetic constants, receptor binding affinities, dose ranges, and biological variability—were derived from previously published, peer-reviewed sources. These were transparently documented and referenced to ensure model reproducibility and scientific integrity. Simulated data were managed in accordance with the FAIR data principles (Findable, Accessible, Interoperable, and Reusable). Model architecture, parameter values, and output variables were version-controlled and annotated to enable complete transparency. All simulations were performed on a secure, audit-traceable platform with computational provenance logs maintained. To ensure reproducibility and analytical rigor:

- Random number generators were seeded deterministically for simulation consistency.
- Data outputs were stored in open formats (e.g., CSV, JSON) compatible with most statistical and scientific software.
- All statistical analyses were conducted using validated, open-source libraries with documented assumptions and confidence intervals.

No personally identifiable information (PII), protected health information (PHI), or biospecimens were involved at any stage of the study. This safeguards against any privacy breaches and maintains strict adherence to ethical standards for digital research. Due to licensing restrictions associated with the proprietary AesthetiSIM™ simulation engine, the full model source code is not publicly available but can be reviewed under a data use agreement for academic, non-commercial research purposes.

## Figures and Tables

**Table 1:** T_80_ Functional Recovery Time Across Groups.

Group	Mean T_80_ (Days)	SD (Days)	95% CI Lower	95% CI Upper	t-Statistic (vs. BoNTA)	*p*-Value	Effect Size (Cohen’s d)
BoNTA + Standard Saline	42.0	4.5	41.88	42.12	–	–	–
BoNTA + Bacteriostatic Saline	35.5	5.0	35.38	35.62	120.11	<0.001	1.38
BoNTA + Normal Saline	41.7	4.7	41.58	41.82	1.42	0.158	0.06

**Table 2:** Time to Initial Reactivation (T_30_).

Group	Mean T_30_ (Days)	SD (Days)	95% CI Lower	95% CI Upper	*p*-Value (vs. BoNTA)	Effect Size (Cohen’s d)
BoNTA + Standard Saline	27.3	4.2	27.18	27.42	–	–
BoNTA + Bacteriostatic Saline	21.8	4.6	21.65	21.95	<0.001	1.26
BoNTA + Normal Saline	26.9	4.3	26.78	27.02	0.261	0.09

**Table 3:** Proportion of Patients Recovered (T_80_) by Timepoint.

Timepoint (Days)	BoNTA + Standard Saline (%)	BoNTA + Bacteriostatic Saline (%)	BoNTA + Normal Saline (%)	Chi-squared Test (BS vs. BoNTA)
Day 14	0.00	0.00	0.00	–
Day 21	0.00	0.28	0.00	3.56 (*p* = 0.059)
Day 30	0.44	13.05	0.57	1452.1 (*p* < 0.001)

**Table 4:** Area Under the Effect Curve (AUEC) Over 42 Days.

Group	Mean AUEC	SD	Δ AUEC vs. BoNTA	Percentage Increase	*p*-Value vs. BoNTA
BoNTA + Bacteriostatic Saline	0.505	0.029	+0.045	+9.7%	<0.001
BoNTA + Normal Saline	0.465	0.026	+0.005	+1.2%	0.089

**Table 5:** BoNTA–SNAP-25 Binding with and without BnOH.

BoNTA Conc. (µM)	Fraction Bound (Normal)	Fraction Bound (BnOH)	Δ Binding (%)
0.1	0.286	0.167	11.90%
0.5	0.667	0.500	16.67%
1.0	0.800	0.667	13.33%
3.0	0.923	0.857	6.59%
10.0	0.976	0.952	2.32%

**Table 6:** Sensitivity Analysis of Model Parameters Influencing T_80_.

Parameter	Sobol Index	Contribution to T_80_ Variance	Interpretation
BoNTA LC degradation rate	0.49	High (Primary driver)	Determines most of the inter-patient variability in recovery
BS-induced membrane permeability increase	0.12	Moderate	Suggests some modulatory effect from BnOH on cytoplasmic efflux
BS effect duration (hours)	0.10	Mild to moderate	Contributes indirectly via exposure window of benzyl alcohol
Endosomal trafficking inhibition	0.08	Mild	Minor contributor to acceleration of vesicle-to-cytoplasm transition
IC_50_ variability (SNARE inhibition sensitivity)	0.06	Minimal	Reflects minor influence of intrinsic synaptic sensitivity
BoNTA dose variation (10–15 U range)	0.03	Negligible	Dose variation within clinical aesthetic range has little effect on recovery

**Table 7:** External Validation Against Clinical Data.

Validation Domain	Simulation Result	Clinical Alignment
BoNTA-only group T_80_ recovery range	Mean T_80_ = 42.0 ± 4.5 days (matches 6–8-week clinical recovery)	Strong concordance with published resolution of frontalis ptosis
Absence of effect in NS group	T_80_ = 41.7 ± 4.7 days; no significant difference from BoNTA-only (*p* = 0.158)	Supports conclusion that needling/saline alone has no effect
BS group early functional shift	T30 accelerated by 5.5 days; 13.05% recovered by Day 30	Consistent with limited partial benefit seen in anecdotal BS reports
Absence of recovery by Day 14 in all groups	0% recovery by Day 14 across all groups	Contradicts rapid reversal claims
Magnitude of BS effect vs anecdotal reports	Effect size smaller than reported 7–14 day “reversals” in uncontrolled case series	Suggests previously reported outcomes likely confounded or misattributed

**Table 8:** Simulation Parameters and Pharmacodynamic Model Assumptions for BoNTA.

Parameter	Value/Range	Source
BoNTA dose (frontalis)	10–15 U	Rullan et al. [1]
Internalization half-life	3–5 h	Rossetto et al. [2]
LC degradation rate (k_deg_)	0.02–0.06 day^−1^	Nigam et al. [3]

**Table 9:** Input Parameter Distributions Used in Global Sensitivity Analysis.

Parameter	Distribution	Notes
LC degradation rate (k_deg_)	Uniform (0.02–0.06)	Reflects biological variability
IC_50_	Log-normal	Range: 0.1–0.5 µM
BS effect duration	Uniform (4–12 h)	Duration of benzyl alcohol action
Muscle output threshold	Fixed (E ≥ 0.8)	Used to define functional recovery

## Data Availability

The original contributions presented in this study are included in the article. Further inquiries can be directed to the corresponding author.
